# Intramammary Angiomatoid Fibrous Histiocytoma, a Rare* EWSR1* Rearranged Mesenchymal Neoplasm in a Previously Unreported Anatomic Location with Review of the Cleveland Clinic Experience

**DOI:** 10.1155/2019/9012878

**Published:** 2019-05-20

**Authors:** F. K. Bruehl, K. L. Cooper, S. E. Kilpatrick, M. D. Weindel, M. Ganea, C. Astbury, E. P. Downs-Kelly, C. D. Sturgis

**Affiliations:** ^1^RJ Tomsich Pathology and Laboratory Medicine Institute, Cleveland Clinic, Cleveland, OH, USA; ^2^Department of Pathology, St. Vincent Charity Medical Center, Cleveland, OH, USA

## Abstract

Angiomatoid fibrous histiocytoma (AFH) is a rare soft tissue tumor that is most commonly reported to arise in the subcutaneous tissues of the upper extremities in adolescents and young adults. At present, the WHO classifies this neoplasm as a tumor of uncertain differentiation. AFH is most often clinically regarded as a tumor of intermediate risk due to low reported rates of recurrence and only rare occurrences of metastases. Its histomorphological hallmarks are a prominent lymphoid cuff surrounding a spindle cell neoplasm with syncytial-appearing cytoplasm. Several variant morphologies have been described. Genetically, the tumor is characterized by translocations involving the* EWSR1* gene in over 90% of cases. A widening range of anatomical locations and morphological variants of AFH has been reported in the literature; however, neither anatomic location nor specific morphologic features have been shown to correlate with clinical/biological behavior. We report a unique case of AFH arising in the parenchyma of the breast. The neoplasm showed the typical histomorphology including a peripheral lymphoid cuff. The lesional cells in this case were found to be immunoreactive with desmin, and a positive* EWSR1* result was confirmed by break-apart fluorescence in situ hybridization testing. To our knowledge, this is the first report of AFH arising in the breast parenchyma of a postmenopausal female.

## 1. Introduction

Angiomatoid fibrous histiocytoma (AFH) is a rare, low-grade, soft tissue tumor most commonly reported in the subcutis or deep dermis of the extremities in persons in the first two decades of life. Currently, the WHO classification of Tumors of Soft Tissue and Bone classifies AFH as a tumor of uncertain differentiation [1]. The entity was first described by Enzinger in 1979 and was termed Angiomatoid “Malignant” Fibrous Histiocytoma [2]. Due to low reported recurrence rates (2-10% locally) and even lower reported risk of metastases (< 1% of cases), its name was changed to AFH, and it is now considered a malignancy with intermediate risk. The classi*c*al morphologic features of AFH include a peripheral lymphoid cuff, a fibrous pseudocapsule, pseudovascular spaces, hemorrhage, hemosiderin, and sheets or short fascicles of bland, epithelioid to spindle cells with histiocytoid morphology.* EWSR1* fusions are reported in over 90% of cases. We report an example of a unique intramammary* EWSR1* rearranged AFH with an intrinsic lymphoplasma cellular infiltrate. To our knowledge, this lesion has not previously been reported in this anatomic location.

## 2. Case Report

A 73-year-old Caucasian woman presented to her primary care physician with a complaint of a painful breast mass. A history of trauma was solicited with the patient reporting that she had been bitten in the breast by a toddler in the weeks preceding her presentation. Per report, no palpable or painful breast abnormality was present prior to the episode of trauma. On physical examination, a well-circumscribed, approximately 2 cm, tender nodule was palpated. The patient was referred to radiology for imaging studies.

**Figure 1:**
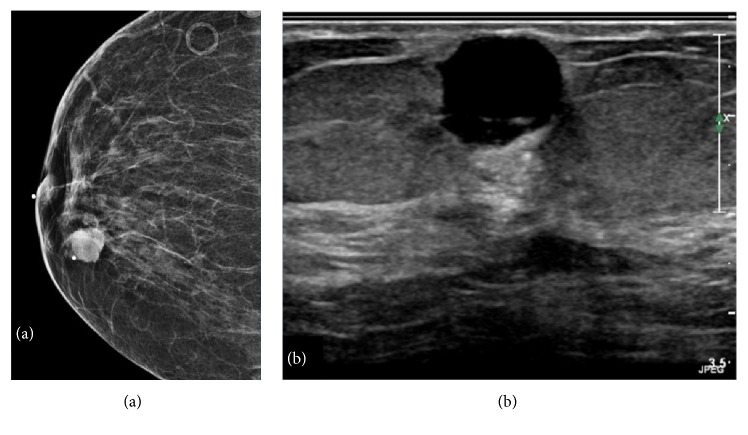
(a) Mammography, left breast: multinodular 19 mm predominantly solid lesion. (b) Ultrasonography, left breast: 19 x 13 x 12 mm complex solid and cystic lesion with internal vascularity located 2 cm from the nipple at 1:00 axis.

**Figure 2:**
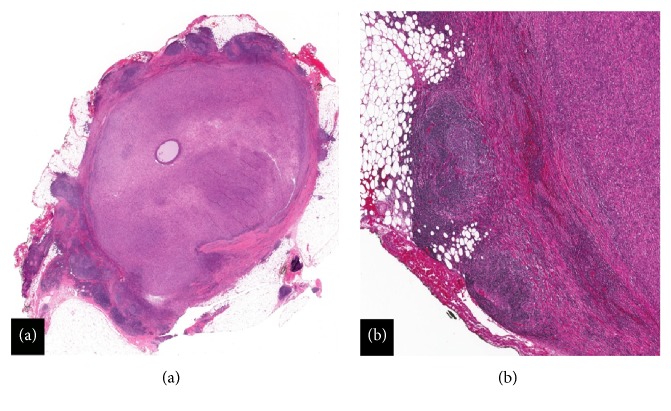
(a) Histology: whole mount section of a well-circumscribed mesenchymal proliferation with a prominent rimming lymphoid cuff, an incomplete eosinophilic fibrous pseudocapsule, and central cyst formation (Hematoxylin & eosin, 2X). (b) Histology: lymphoid cuff with germinal center formation and fibrous pseudocapsule (Hematoxylin & eosin, 20X).

**Figure 3:**
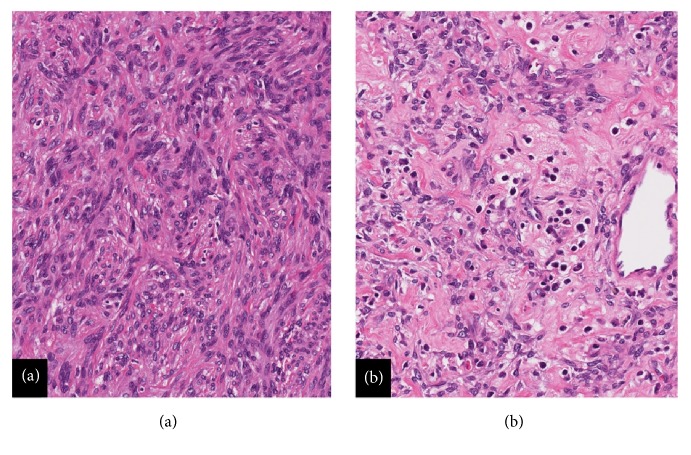
(a) Histology: spindle cell neoplasm with sheet-like growth and syncytial cytoplasmic appearance (Hematoxylin & eosin, 20X). (b) Histology: scattered intrinsic lymphocytes and plasma cells (Hematoxylin & eosin, 20X).

**Figure 4:**
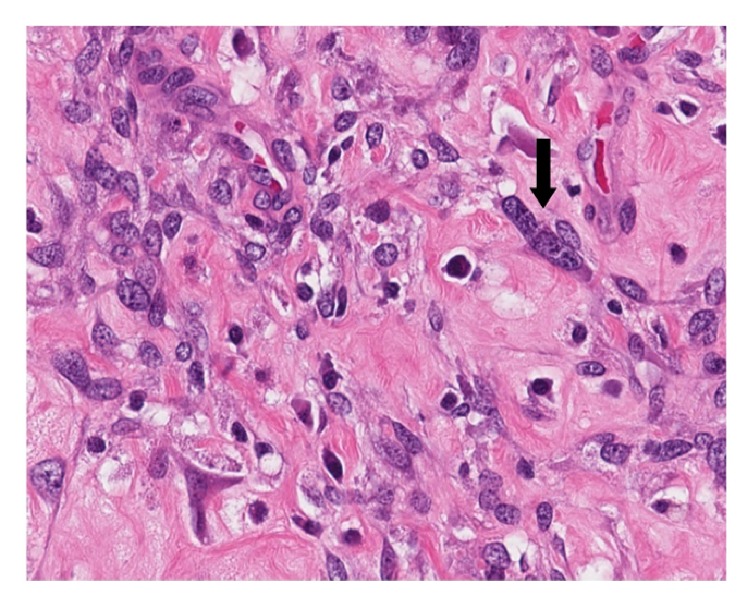
Histology: focal nuclear pleomorphism with scattered single cells showing moderate anisonucleosis, arrow indicating a larger rectangular nucleus with 4X the nuclear volume than adjacent tumor cell nuclei (Hematoxylin & eosin, 40X).

Mammography and ultrasonography of the left breast were performed (Figures 1(a) and 1(b)). Imaging studies confirmed a 19 x 13 x 12 mm solid and cystic mass with internal vascularity. This lesion was located 20 mm from the nipple at the 1:00 location. The interpreting radiologist classified the lesion as BI-RADS 4—suspicious abnormality. Surgical consultation was sought, and the patient underwent excision. A 50 x 40 x 25 mm portion of fibrofatty breast tissue was received in pathology. Cross sectioning revealed a well-circumscribed solid nodule measuring 16 mm in greatest dimension. Intraoperative frozen section histologic studies confirmed a mesenchymal neoplasm with specific classification deferred to permanent sections. Histological assessment of paraffin-embedded tissue showed a well-circumscribed mesenchymal lesion (Figure 2(a)) comprised of spindle cells surrounded by a fibrous pseudocapsule with a prominent pericapsular lymphoid cuff focally containing germinal centers (Figure 2(b)). The lesional spindled cells were present in sheet-like expanses with a syncytial appearance (Figure 3(a)). Scattered intrinsic lymphocytes and plasma cells were noted (Figure 3(b)). The lesional cells had open chromatin with predominantly single nucleoli. Moderate cellular pleomorphism was present with random nuclei enlarged to a ratio of 4:1 in comparison to the majority of lesional cells (Figure 4). Mitoses were present at a rate of 2 to 3 per 10 high-power fields.

**Figure 5:**
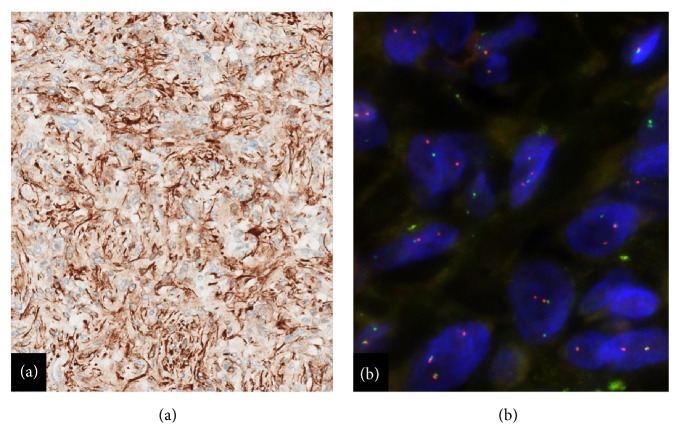
(a) Immunohistochemistry: desmin, lesional cells diffusely immunoreactive (20X). (b) Fluorescence in situ hybridization with a dual-color break-apart probe flanking the* EWSR1* gene on chromosome 22q12. A separate red and green signal indicates a rearrangement involving* EWSR1* and is identified in the lesional cells (100X).

A battery of immunohistochemical tests was performed. The lesional cells were found to be strongly immunoreactive for desmin (Figure 5(a)) and focally immunoreactive for p63. The lesional cells were nonreactive for all other markers, including CD34, CK5/6, ALK-1, and STAT6. The sample was then triaged for cytogenomics testing, and break-apart fluorescence in situ hybridization (FISH) studies [Vysis LSI* EWSR1* (22q12) Dual-Color Probe, Abbott Molecular, Des Plaines, IL] confirmed an* EWSR1* gene rearrangement (Figure 5(b)). The combined histomorphologic, immunohistochemical, and molecular findings together allowed for a definitive diagnosis of an AFH.

## 3. Discussion

**Figure 6:**
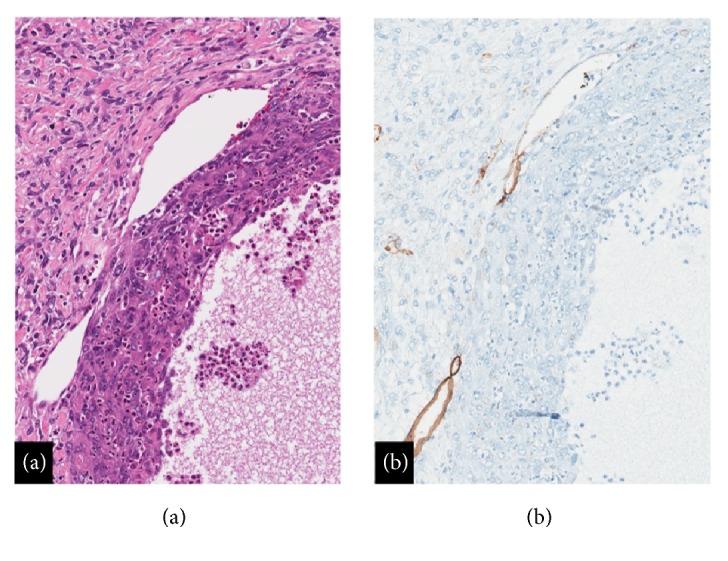
(a) Histology: central cystic pseudoangiomatous space with acute inflammation and adjacent compressed small thin-walled vessels (Hematoxylin & eosin, 20X). (b) Immunohistochemistry: CD34, immunoreactivity present in thin-walled vascular spaces with absence of endothelial marking in larger pseudoangiomatous cystic cavity (20X).

A lesion of the breast in a 73-year-old female with the imaging findings shown in Figure 1 raises suspicion for primary breast carcinoma. With the pertinent clinical history of trauma, other processes such as hematoma, abscess, and fat necrosis also enter the differential diagnosis. The imaging finding of internal cystic change raises suspicion for other lesions such as fibrocystic mastopathy, phyllodes tumor, papilloma, and metastasis. Surgical excision was performed without intervening core needle biopsy or fine needle aspiration, and intraoperative frozen section examination was performed. Frozen section histology did not allow for a specific diagnosis. On permanent section histologic studies, the lesion demonstrated classic histological features of AFH including a ringing pericapsular lymphoid cuff surrounding a monotonous spindle cell proliferation. A central cystic space rimmed by acute inflammation and containing cellular debris was identified, correlating to the radiological finding of intrinsic cystic changes (Figure 6(a)). This cystic “pseudoangiomatous space” is another histological hallmark and eponymous feature of AFH. The absence of CD34 staining in the cells lining the cystic space fits the interpretation of a “pseudoangiomatous,” rather than of a true vascular space, although CD34 is not an entirely sensitive or specific marker for endothelial differentiation (Figure 6(b)).

Several reports have described a spectrum of morphological patterns and clinical characteristics for AFH [3, 4]. Reported morphological types of AFH have included sclerotic, pleomorphic, eosinophilic, perineurioma-like, and myxoid. Various types of AFH have also been described to contain intrinsic areas with edema, clear cell change, rhabdomyoblast-like cells, and calcifications [5, 6]. The current example showed a nonspecific sheet-like growth pattern with scattered mitoses and dispersed single cell nuclear pleomorphism. In addition, this intramammary AFH showed a dispersed population of intratumoral lymphoplasmacytic mononuclear inflammatory cells sprinkled amongst the spindled tumor cells, appearing distinct from the lymphoid cuff.

A prior peer-reviewed manuscript from the Cleveland Clinic described 27 cases of AFH that were retrieved from files spanning the time frame from 1980 through 2012 [3]. Since 2012, 13 additional cases of AFH, including the current case, have been recorded (Table 1). The age range for all patients extended from 8 years to 73 years with the current case being the outlier at 73 years. This shows that while AFH are commonly thought of as occurring in adolescents, they can also be found in older adults. The majority of these lesions were excised from the extremities, with rare cases reported on the cheek, the back, and only the current case from the adult female breast, further highlighting the rarity of the present case. Fluorescence in situ hybridization was performed in seven of the 13 more recent cases, with* EWSR1* rearrangements documented in all of the tested cases.

In the 2012 case series from Bohman et al., AFHs were reported to be immunoreactive with CD68 (65%), EMA (65%), and desmin (56%). Of note, 100% (9/9) of AFH cases tested since 2012 at Cleveland Clinic were found to be immunoreactive with desmin IHC, highlighting the usefulness and potential sensitivity of this marker. 60% (3/5) of AFH cases since 2012 showed immunoreactivity with EMA. It has been suggested that the “unusual combination” of EMA and desmin can raise suspicion for a diagnosis of AFH in an appropriate histologic context [7]. This rare immunophenotype is also seen in the clinically and histologically distinct entity of desmoplastic small round cell tumor, which interestingly also shows an* EWSR1* rearrangement, although most commonly with a different partner, forming the pathognomonic* EWSR1-WT1* fusion gene [8, 9]. Rhabdomyosarcoma may be considered as a lymph node metastasis in cases with spindled cells composing a nodule resembling a replaced lymph node. While smooth muscle actin is negative in both rhabdomyosarcoma and AFH, other markers of muscular differentiation such as myogenin and MyoD1 are only negative in AFH, helping to avoid this diagnostic pitfall [10].

Of particular interest to practitioners of breast pathology, mammary-type myofibroblastomas are benign mesenchymal neoplasms comprised of sheets of myofibroblasts, and these tumors are often immunoreactive with desmin [11, 12]. Occasional examples of myofibroblastoma have also been reported to have myxoid features and cellular pleomorphism, as can be seen in some examples of AFH [13]. Importantly, myofibroblastomas lack a lymphoid cuff and often contain intrinsic islands of adipose tissue. In addition, CD34 immunoreactivity is seen in most myofibroblastomas, while CD34 is not expressed in AFH [11, 12, 14]. Absence of vascular markers also helps to exclude Kaposi sarcoma and angiosarcoma, and absence of staining for S100 and cytokeratin can exclude the differential diagnoses of melanoma and sarcomatoid/metaplastic carcinoma.

AFH was recently shown to have a high rate of ALK expression using various antibodies [15]. ALK immunoreactivity (especially in small biopsies) may result in confusion between AFH and inflammatory myofibroblastic tumor. Immunohistochemistry is therefore useful but has pitfalls and AFH lesions are not identifiable by a single specific immunohistochemical marker or even a combination of markers, and molecular or cytogenetic ancillary studies may be of diagnostic value.

Demonstration of specific gene rearrangements may support the diagnosis of AFH. The first group to describe a genetic abnormality in AFH was Waters et al., who identified two cases which contained t(12;16)(q13.12;p11.2), involving* FUS* and* ATF1* genes [16]. Subsequent studies showed that the most common aberration is t(2:22)(q33.3;q12.2) forming the* EWSR1-CREB1* fusion gene which is present in > 70% of cases of AFH [17]. Additionally, t(12:22)(q13.12;q12.2) resulting in an* EWSR1-ATF1* fusion gene has also been documented for AFH [17, 18]. Rearrangements of* EWSR1* have been described in a variety of other tumors.* EWSR1-CREB1* and* EWSR1-ATF1* fusions are also present in clear cell sarcoma. While the molecular alteration in CCS and AFH can be identical, CCS is characterized by nested epithelioid to spindled cells with an immunohistochemical expression of melanocytic markers, thus highlighting the need for skilled histomorphologic assessment of these lesions, regardless of the result and comprehensiveness of molecular and cytogenetic testing. For the purpose of this case report, a break-apart probe for the* EWSR1* gene on chromosome 22q12.2 was evaluated by fluorescence in situ hybridization and showed a positive rearrangement of the interrogated gene locus. The fusion partner was not further characterized. While a positive* EWSR1* FISH is not specific for AFH, this result, in combination with morphology and immunohistochemistry, is supportive of the diagnosis.

## 4. Conclusion

Classical morphologic features of AFH were present in this case and are the most important criteria to correctly diagnose AFH. Immunohistochemical studies as well as molecular confirmation of a positive* EWSR1* rearrangement corroborate the diagnosis. To our knowledge, this is the first report of AFH arising in the substance of the adult female breast, expanding the spectrum of anatomical locations in which AFH occurs.

## Figures and Tables

**Table 1:** Features of AFH cases from Cleveland Clinic (02/2012 through 02/2019).

	Age	Sex	Location	Desmin IHC Result	EMA IHC Result	EWSR1 FISH Result
1	58	F	Hand	Positive	Negative	Positive
2	68	F	Foot	Positive	Positive	Positive
3	25	F	Cheek	Positive	Not performed	Not performed
4	54	F	Arm	Not performed	Not performed	Not performed
5	19	M	Popliteal	Positive	Not performed	Positive
6	11	F	Upper Arm	Positive	Negative	Not performed
7	8	M	Thumb	Positive	Positive	Positive
8	10	M	Elbow	Not performed	Not performed	Positive
9	40	M	Back	Positive	Positive	Not performed
10	14	M	Popliteal	Not performed	Not performed	Positive
11	48	M	Upper Arm	Positive	Not performed	Not performed
12	11	F	Forearm	Not performed	Not performed	Not performed
13	73	F	Breast	Positive	Not performed	Positive
